# Salvianolic acids: small compounds with multiple mechanisms for cardiovascular protection

**DOI:** 10.1186/1423-0127-18-30

**Published:** 2011-05-11

**Authors:** Jennifer Hui-Chun Ho, Chuang-Ye Hong

**Affiliations:** 1 Graduate Institute of Clinical Medicine, Taipei Medical University, Taipei, Taiwan; 2 Department of Ophthalmology, Wan Fang Hospital, Taipei Medical University, Taipei, Taiwan; 3 Center for Stem Cell Research, Wan Fang Hospital, Taipei Medical University, Taipei, Taiwan; 4 Department of Medicine, Wan Fang Hospital, Taipei Medical University, Taipei, Taiwan

## Abstract

Salvianolic acids are the most abundant water-soluble compounds extracted from Radix *Salvia miltiorrhiza *(Danshen). In China, Danshen has been wildly used to treat cardiovascular diseases for hundreds of years. Salvianolic acids, especially salvianolic acid A (Sal A) and salvianolic acid B (Sal B), have been found to have potent anti-oxidative capabilities due to their polyphenolic structure. Recently, intracellular signaling pathways regulated by salvianolic acids in vascular endothelial cells, aortic smooth muscle cells, as well as cardiomyocytes, have been investigated both *in vitro *and *in vivo *upon various cardiovascular insults. It is discovered that the cardiovascular protection of salvianolic acids is not only because salvianolic acids act as reactive oxygen species scavengers, but also due to the reduction of leukocyte-endothelial adherence, inhibition of inflammation and metalloproteinases expression from aortic smooth muscle cells, and indirect regulation of immune function. Competitive binding of salvianolic acids to target proteins to interrupt protein-protein interactions has also been found to be a mechanism of cardiovascular protection by salvianolic acids. In this article, we review a variety of studies focusing on the above mentioned mechanisms. Besides, the target proteins of salvianolic acids are also described. These results of recent advances have shed new light to the development of novel therapeutic strategies for salvianolic acids to treat cardiovascular diseases.

## Introduction

**Figure 1:**
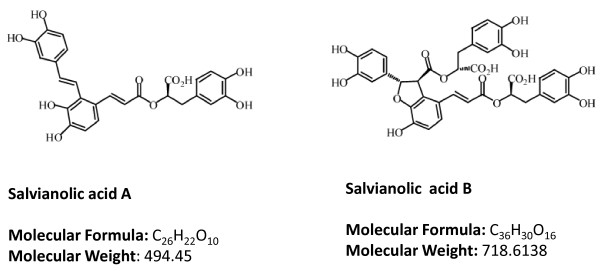
**Chemical structure of salvianolic acid A (Sal A) and Sal B**. More than eighteen components can be identified in Radix *S. miltiorrhiza*. Sal B is the most abundant while Sal A is the most potent water-soluble phenolic component in Radix *S. miltiorrhiza*.

Salvianolic acid is one of the bioactive compounds of *S. miltiorrhiza *BGE extracted from the root of *S. miltiorrhiza*, commonly named "Danshen" in China. According to traditional Chinese medicine, Danshen can be used to promote blood flow and to resolve blood stasis. Therefore, it is wildly prescribed to patients with angina pectoris, hyperlipidemia, and acute ischemic stroke [1-3]. Using chromatographic fingerprinting method and mass spectrometry, there are more than eighteen components in Radix *S. miltiorrhiza*. They can be classified as water-soluble (hydrophilic) phenolic compounds and nonpolar (lipid-soluble) diterpenoidal compounds [4,5]. Salvianolic acids are the main water-soluble compound in *S. miltiorrhiza*. Among salvianolic acids, Sal A and Sal B are the most abundant components. The structures of salvianolic acids are shown in figure 1.

The bioavailability, pharmacodynamics as well as pharmacokinetics of salvianolic acids have been investigated. Due to first-order absorption, Sal B reaches the maximum plasma concentration within 0.5-1 hour and could be detected up to 180 minutes after oral administration [6,7], and which undergoes hepatobiliary excretion [8]. In conscious and freely moving rat, Wu et al. demonstrates that Sal B shows the linearity over a plasma concentration range of 0.5-200 μg/ml and 83.78 ± 10.5% of plasma protein binding rate. By intravenous injection, 100 mg/kg Sal B reaches the maximal plasma concentration (*C*_max_) around 910 μg/ml, and the half life (*t*_1/2_) of Sal B is around 105 minutes [7]. For oral administration, the *C*_max _of 500 mg/kg Sal B is 1.5 μg/ml while 100 mg/kg Sal A is only 308 ng/ml; the *t*_1/2 _is around 248 minutes for Sal B and 3.29 hours for Sal A [7,9]. The oral bioavailability of Sal B in a conscious rat is calculated to be 2.3% [7], which is higher than rats under general anesthesia reported by Zhang et al [10].

Although the therapeutic potential of salvianolic acids on hepatic protection [11,12], neural protection [13,14], and cancer treatment [15-17] have been proposed in recent years, the greatest clinical impact of salvianolic acids is cardiovascular protection. In the past few years, mechanism(s) of how salvianolic acids regulate endothelial cells, vascular smooth muscle cells and cardiomyocytes have been investigated. In this article, we summarize results of these studies on the cardiovascular protective effect of salvianolic acids and elucidate the multiple mechanisms of these small compounds in terms of reactive oxygen species (ROS) scavenging ability, leukocyte-endothelial adhesion regulation, inflammation inhibition and immune-modulation. In addition, intracellular signaling pathway regulated by salvianolic acids as well as putative proteins targeted by salvianolic acids are described in this article.

## Cardiovascular Peotection of Salvianolic Acids

### A.	Salvianolic Acids Serve As Potent Ros Scavengers During Cardiovascular Injury

Due to their polyphenolic structure, salvianolic acids are thought to be free radical scavengers. Indeed, both Sal B and Sal A show their high radical scavenging capacity measured by neutralizing free radicals assays such as DPPH radical scavenging test or ABTS assay [18-20]. Liu et al reported that seven phenolic compounds isolated from *S. miltiorrhiza *inhibited lipid peroxidation of rat liver microsomes induced by iron/cysteine and vitamin C/NADPH and the hemolysis of rat erythrocytes induced by H_2_O_2 _*in vitro *[21]. It was found that Sal A was the most potent antioxidant among the salvianolic acids. However, Sal B was thought to have much more commercial value for the food and medicine purposes due to the containment of the highest amounts in *S. miltiorrhiza *[4,18,21]. Sal B exhibited higher scavenging activities than vitamin C against HO^·^, O2^·-^, DPPH radicals, and ABTS radicals. However, their iron chelating and H_2_O_2 _scavenging activities were lower than vitamin C [20].

Salvianolic acids were not only demonstrated to have antioxidant activity *in vitro*, but also been proven to act as cardiovascular protectors *in vivo*. Using ischemia-reperfusion injury model of an isolated rat heart, it was demonstrated that Sal A lowered the ventricular fibrillation rate, decreased cellular LDH leaking and reduced lipid peroxidation in damaged cardiac tissue [22]. Wu and Hong et al reported that feeding with 5% water-soluble extract of *S miltiorrhiza *which contained Sal B significantly lowered plasma cholesterol level, reduced endothelial damage and the severity of atherosclerosis in diet-induced hypercholesteremic rabbits. The cardiovascular protection potential of Sal B was contributed by its ROS scavenging ability. Sal B-treated LDL exhibited vitamin E-binding ability and was resistant to Cu^2+^-induced oxidation [23]. Moreover, intravenous administration of Sal A (0.3-3 mg/kg) significantly attenuated isoproterenol-induced cardiac dysfunction and myocardial injury, and improved mitochondrial respiratory function in rat with isoproterenol-induced myocardial infarction [24].

It is known that increase in oxidative stress induced the proliferation of aortic smooth muscle cells. Recently, salvianolic acids were found to inhibit the proliferation of rat aortic smooth muscle A10 cells stimulated by homocysteine, an oxidative stress factor. Elucidation of proteomic changes by two-dimensional electrophoresis coupled with MALDI-TOF mass spectrometry revealed that the inhibitory effect of the salvianolic acid on homocysteine-induced A10 cell proliferation was via the PKC/p44/42 MAPK dependent pathway [25]. Interestingly, salvianolic acid treatment reduces the carbonylation of specific cytoskeleton and chaperone proteins such as vimentin, α4-tropomyosin and GRP75, and lead to phenotype transformations in the rat A10 cells [25].

Apart from what have been mentioned above, salvianolic acids have been reported to protect cardiomyocytes from drug-induced toxicity due to its ROS scavenging ability. It was noted that Sal A converted HO^· ^generated by electron transfer from adriamycin semiquinone radicals to H_2_O_2 _on adriamycin-induced mitochondrial toxicity of rat heart in a dose-dependent manner [26]. In mice with doxorubicin-induced cardiotoxicity, salvianolic acids (containing 64.92% Sal B, 40 mg/kg/day for 3 days) also protected myocardium through reducing oxidative stress [27].

### B.	Salvianolic Acids Inhibit Leukocyte-Endothelial Cell Adherence

Leukocyte attachment, migration as well as adhesion molecule expression on arterial endothelial cells all are important steps in the development of early atherosclerosis. A series of studies on salvianolic acids regulating leukocyte-endothelial cell adherence have been undertaken. It is well-established that endothelial-leukocyte adhesion molecules on aortic endothelial cells can be induced by TNF-α. Sal B was found to attenuate VCAM-1 and ICAM-1, but not E-selectin expression, in a dose-dependent manner (1-20 μg/ml), in TNF-α-treated HAECs. These effects were associated with its anti-inflammatory property through inhibiting the activation of NFkB pathway triggered by TNF-α [28].

Using Evans Blue dye-labeled bovine serum albumin to investigate the alteration of permeability in HUVECs caused by Sal B (20-50 μg/ml), it was found that Sal B reduced the permeability and attenuated the disorganization of VE-cadherin induced by TNF-α in these cells. The effect was attributed to the reduction of VEGF protein expression as a result of modulation of the ERK pathway [29]. Loss of cell-cell adhesion junctions also increased endothelial permeability. Dang et al demonstrated the protective effect of Sal B on TNF-α-mediated disorganization of endothelial cell junctions through attenuating tyrosine phosphorylation of cell junction proteins such as VE-cadherin and β-catenin. Results of immuno-precipitation studies indicated that Sal B prevented β-catenin disassociation from the cytoskeleton in TNF-α-treated HUVECs [30].

Zhou et al reported that treatment with Sal B (0.05 and 0.15 μM) significantly inhibited PAI-1 gene expression in the first 18 hours when HUVECs were exposed to TNF-α. It was further demonstrated that NFkB and ERK-AP-1 pathways were possible targets of Sal B in regulating TNF-α-stimulated PAI-1 production in HUVECs [31].

### C.	Salvianolic Acids Inhibit Inflammation And Regulation of Metalloproteinaseses Expression During Cardiovascular Injury

Synthesis and release of inflammatory cytokines from vascular smooth muscle cells is an important contributor to the pathogenesis of atherosclerosis. Chen et al and Lin et al reported respectively that genetic expression of COX-2 and protein expression of MMP-2 and MMP-9 in LPS-treated HASMCs could be inhibited by Sal B via the suppression of ERK1/2 and JNK phosphorylation, reduction of PGE2 production and NADPH oxidase activity [32,33]. In ApoE-deficient mice fed with high cholesterol diet, supplementation with 0.3% of Sal B protected mice from atherosclerosis by reducing the thickness of intima, which was accompanied by a significant reduction of COX-2, MMP-2 and MMP-9 expression [32,33].

Sal B not only inhibited MMP-2 activation induced by LPS, but also inhibited MMP-2 activation induced by TNF-α, angiotension II and H_2_O_2_. It has been demonstrated that Sal B inhibited TNF-α, angiotension II and H_2_O_2_-induced MMP-2 mRNA, protein expression, and gelatinolytic activity in HASMCs in a concentration-dependent manner (0.1-10 μM), which was through the inhibition of NADPH oxidase-dependent ROS generation [34].

In experimental myocardial infarction in rat, Jiang et al reported that administration of salvianolic acids significantly decreased infarct size, improved left ventricular function and decreased myocardial malondialdehyde levels compared with the control group. The cardioprotection of salvianolic acids against infarct-induced left ventricle remodeling was significantly contributed by the down-regulation of MMP-9 mRNA expression level and its activity at the infarct area [35]. With molecular modeling, in-gel gelatin zymography and enzymatic analysis, Jiang et al demonstrated that Sal B (from 0-70 μM) bound to MMP-9 at catalytic domain and functioned as a competitive inhibitor of MMP-9 [36].

### D.	Salvianolic Acids Regulate Kinase Activity and are Potential Immunomodulators

Recently, the putative protein targets of salvianolic acids have been investigated [37]. In addition to MMP-9 binding affinity [36], salvianolic acids regulate intracellular kinase-associated signaling pathway [28,29,31-34], indicating that salvianolic acids interact with phosphotyrosine or phosphoserine/threonine-binding domain [38,39]. With binding affinity assay and molecular modeling prediction, Sperl et al reported that Sal A and Sal B were inhibitors of the protein-protein interaction mediated by SH2 domains of Src-family kinases Src and Lck. The potency of Sal A and Sal B (from 0-100 μM) binding to Src and Lck were higher than rosmarinic acid, a nature product known as a Lck SH2 domain inhibitor [40]. Since Lck is a T cell-restricted Src family protein tyrosine kinase and is crucial in TCR-mediated signaling pathway, the activity of rosmarinic acid against Lck SH2 domain has been used experimentally as an immune-suppressive agent [41] and becomes a focus of cancer drug discovery [42].

Recently, Wang et al reported that using an ELISA-like HTS assay, Sal B and rosmarinic acid were found to be active compounds showing high affinity against CD36, a high affinity receptor for oxLDL, thus prevented oxLDL from macrophage uptake [43]. Since both Sal A and Sal B shared the core structure of rosmarinic acid [40], the high affinity to SH2 domains of Src-family kinases and CD36 suggested the role of immune modulator in the cardiovascular protective effect of salvianolic acids.

## Conclusion

Salvianolic acids, which contain polyphenolic structure, are potent antioxidants. Salvianolic acids reduce intracellular as well as intravascular oxidative stress, which protects endothelial cells, arterial smooth muscle cells, cardiomyocyte, and LDL form free radical damage and peroxidation. In addition, salvianolic acids attenuate endothelial-leukocyte adhesion molecules expression on vascular endothelial cells through regulating intracellular kinase activity. Such kinase-associated signaling pathway inhibition by salvianolic acids also contributes their anti-inflammation effect. Salvianolic acids possess strong affinity to bind MMP-9, SH2 domain of the Src-family kinases and CD36, which inhibits protein-protein interaction. For clinical application, intravenous injection rather than oral administration of such a water-soluble compound is more easily to reach the therapeutic plasma concentration. Taken together, the cardiovascular protective effect of salvianolic acids is mediated through multiple molecular mechanisms. Such unique property makes salvianolic acids excellent candidates for future development of cardiovascular protective agents.

## List of abbreviations

*S. miltiorrhiza = Salvia miltiorrhiza*; Sal A = salvianolic acid A; Sal B = salvianolic acid B; ROS = reactive oxygen species; DPPH = 1,1-diphenyl-2-picrylhydrazyl; ABTS = 2,2-azino-bis-(3-ethylbenzothiazoline-6-sulfonic acid; NADPH = nicotinamide adenine dinucleotide phosphate; H_2_O_2 _= hydrogen peroxide; HO^·^= free hydroxyl radicals; O2^·-^= superoxide anion radicals; LDH = lactate dehydrogenase; LDL = low-density lipoprotein; MALDI-TOF = matrix assisted laser desorption ionization- time of flight; PKC = protein kinase C; MAPK = mitogen-activated protein kinase; α4-tropomyosin = alpha-4-tropomyosin; GRP75 = glucose regulated protein 75; TNF-α = tumor necrosis factor-alpha; VCAM-1 = vascular adhesion molecule-1; ICAM-1 = intercellular cell adhesion molecule-1; E-selectin = endothelial cell selectin; HAECs = human aortic endothelial cells; NFkB = nuclear factor kappa B; HUVECs = umbilical vein endothelial cells; VE-cadherin = vascular endothelial cadherin; VEGF = vascular endothelial growth factor; ERK = extracellular signal-regulated kinase; β-catenin = beta-catenin; PAI-1 = plasminogen activator inhibitor type 1; AP-1 = activating protein-1; COX-2 = cyclooxygenase-2; MMP = metalloproteinases; LPS = lipopolysaccharide; HASMCs = human aortic smooth muscle cells; JNK = c-Jun N-terminal kinases; PGE2 = prostaglandin E2; ApoE = apolipoprotein E; SH2 = Src Homology 2; Lsk = lymphocyte-specific protein tyrosine kinase; TCR = T cell receptor; ELISA = enzyme-linked immunosorbent assay; HTS = high-throughput screening; oxLDL = oxidized low-density lipoprotein; CD = cluster of differentiation.

## Competing interests

The authors declare that they have no competing interests.

## Authors' contributions

JH carried out the design, acquisition, analysis and interpretation of data, drafting the manuscript. CY had contributed to conception, design and critical version of important intellectual content and final approval of the manuscript.

## Authors' information

Dr. Jennifer Hui-Chun Ho is the Director of Center for Stem Cell Research and the Deputy Director of Medical Research and Education at Wan Fang Hospital, Taipei Medical University, and is also an Assistant Professor at Graduate Institute of Clinical Medicine, Taipei Medical University. Her main research theme is translational research of stem cells, especially stem cell transplantation.

Prof. Chuang-Ye Hong is the Superintendent of Wan Fang Hospital, Taipei Medical University and also a consultant cardiologist at Wan Fang Hospital. Prof. Hong is an expert in pharmacological research of medical herbs and cardiovascular diseases. Prof. Hong was the Director of Institute of Traditional Medicine at National Yang-Ming University from 1992 to 1997, when he led a research team working on translational research of traditional Chinese medicine, especially Danshen and magnolol.
